# Staying Connected and Prepared for Collegiate Athletic Competitions During the COVID-19 Pandemic

**DOI:** 10.3389/fspor.2021.663918

**Published:** 2021-03-12

**Authors:** Allie Reynolds, Alireza Hamidian Jahromi

**Affiliations:** ^1^Princeton University, Princeton, NJ, United States; ^2^Plastic and Reconstructive Surgery Department, Rush University Medical Center, Chicago, IL, United States

**Keywords:** COVID-19, collegiate athletics, healthcare, competition, mental health, connectedness, visualization

## Introduction

“*Drastic times call for drastic measures”*—Hippocrates.

The COVID-19 pandemic has had vast and lasting impacts on virtually every aspect of life. Without question, the sports world today looks very different than it looked a year ago. Professional sports leagues adapted to the pandemic by implementing bubbles and strict testing protocols (Dove et al., 2020), but college sports have been slower to adapt. All spring college athletes lost a full season of competition when National Collegiate Athletic Association (NCAA) leagues canceled sporting events in March 2020 and the same thing happened for some fall and winter athletes. With many gyms and other training facilities closed due to the regional lockdown regulations due to the pandemic around the country, it is difficult for college athletes to train on their own and be physically prepared for a collegiate season. More importantly, being separated from teammates has long term impacts on team culture and both mental and physical health. The question remains: how do college athletes stay connected with teammates and prepare for competitions despite being spread out across the country or even the world?

## Importance of Mental Health and Team Culture

Part of being successful as a member of a “team sport” involves being on the same page as your teammates, something that develops over time and with group practice. Finding ways to virtually work together as a team during the pandemic can help develop a sense of identity and community for teammates, but the pandemic could also isolate individuals and drive them apart (Ball, 2020). For college athletes who had their seasons in 2020 canceled, they lost valuable time for training together and forming bonds in the offseason can help them be successful during the upcoming seasons. This puts them behind when practices are allowed to start up, meaning that they have to quickly form bonds and learn how to work together and risk rushing the formation of these relationships rather than allowing them to form naturally. Some teams could be at a disadvantage if they take longer to do this prior to the resumption of competitions, but others could use this to their advantage if they quickly learn to work together. Successful teams could be defined as those that work best together and the extra stress of doing so in a pandemic could either force teammates together or drive them apart (Ball, 2020). As a result, leadership becomes even more important in these instances. In a time of uncertainty and high stress, such as during the pandemic, strong leaders could elevate a previously poor performing team by creation of harmony between teammates and management group and through encouraging collaboration and connections (Hoyt, 2016). While there is a risk that relationships between teammates might feel “manufactured” and “rushed,” it's important that these relationships, even if initially felt artificial, are fostered for the sake of “positive team culture” and meaningful preparation for competition.

Mental health is an underdiscussed topic in the world of collegiate athletics and is even more important as the pandemic forces social distancing and quarantine measures to be enacted. Research shows a causal relationship between social connectedness and mental health (Kawachi and Berkman, 2001; Cruwys et al., 2014; Perkins et al., 2015), meaning that maintaining connections among athletes is crucial for mental health purposes. Although these connections can't happen in person and through the conventional platforms i.e., group training, etc. during the pandemic, there are a variety of online platforms that college programs can use to keep their athletes connected, including Zoom, Cisco WebEx, TeamBuilder, TeamWorks, and countless other social media platforms (Huyghe et al., 2020; Jukic et al., 2020). College athletes can use these online platforms to stay connected with teammates, coaches, and other staff members for motivation and inclusivity purposes (Hayes, 2020; Leng and Phua, 2020). While enhancing social connectedness is a meaningful way of improving mental health for team sport athletes when forced apart during the pandemic, this time apart should also be used to promote other aspects of mental health as well as keeping up the physical preparedness. Athletes should be using the time that would normally be spent training in gyms or other facilities to work on their mental health and stress management techniques. One way of doing so is by increasing the amount of sleep athletes are getting every night. It's important that athletes are getting enough sleep every night under normal circumstances, but especially so during the pandemic. With the media constantly distributing new information about the state of the pandemic, it can be overwhelming and anxiety-provoking at times.

It's been previously shown that poor sleep quality is associated with poorer mental health (Andreato et al., 2020) and less sleep is known to be associated with increased stress levels (Jukic et al., 2020; Wackerhage et al., 2020), showing that sleep should be a priority for all athletes. Sleep deprivation impairs physical and cognitive performance in athletes (Fullagar et al., 2015; Mejri et al., 2016) and can alter someone's psychological state, likely impacting athletic performance (Scott et al., 2006). Using this unprecedented time to improve sleep habits could have long-term benefits for college athletes. This becomes even more important in the event that an athlete is in a quarantine or confinement period, as this time could be viewed as a period over which the athlete has little control (Halabchi et al., 2020). Previous research shows that health impacts could be related to the duration of quarantine as longer periods are associated with poorer mental health (Brooks et al., 2020). Reinforced virtual connections and improved sleep habits established prior to quarantine or isolation periods could help to mediate mental health problems in these instances.

## Physical Activity Alternatives

Since the virus is transmitted from direct person-to-person contact and social distancing has been promoted as a way to decrease transmission, it is important for everyone (including athletes) to stay home whenever possible. This makes it difficult for athletes to maintain their physical fitness, especially with many gyms being closed during the pandemic, and collegiate programs have needed to be innovative in finding solutions to this problem. Practicing as a group with teammates and coaches in person has shifted to online platforms to encourage training at home (Wagner, 2011; Park et al., 2020). Relatedly, video analysis has always been a productive way for athletes to analyze their skillsets and gather feedback from others, and it is even more relevant during the pandemic (Hayes, 2020; Tayech et al., 2020). Other researchers suggest using the time physically apart from teammates to improve visual and kinesthetic motor imagery skills, especially for athletes who don't have access to equipment or facilities (Moran et al., 2012; Ridderinkhof and Brass, 2015). By visualizing their performance, athletes can practice motor actions in their mind without performing actual motor actions.

It has even been shown that visualizing muscle contractions for certain muscle groups can improve strength solely through nervous system adaptations (Folland and Williams, 2007). Importantly, it's necessary for college athletes to separate their negative perceptions of being able to physically workout less with poorer performance in the future (Clemente-Suárez et al., 2020). College athletes also should keep in mind that the pandemic isn't just affecting their workouts, it's impacting every athlete they could be competing against in the same sense. Adaptability and improvising are key in managing workouts during this period and staying ready for future competitions. Using online platforms for video training and performance visualization are very manageable ways to do just that.

## Discussion

Being a member of a team is an enormous part of a college athlete's identity. When the pandemic began and collegiate competitions were canceled, student athletes lost much more than just a season; they lost an important part of who they are. Balancing being both a student and an athlete in college can be compared to having a full-time job and studying at the same time. Since most students were sent home at the beginning of the pandemic, student athletes missed pieces of both the “student” role and the “athlete” role. From the perspective of the universities, student athletes are valuable to have on campus because they bring in revenue from competitions, raise university rankings, and provide extra motivation for other students. Without student athletes on campus and sports competitions occurring, many colleges and universities are struggling. Some educational institutions, even very reputable and financially stable ones, have even gone to extreme lengths as to permanently cut a number of sports due to loss of revenue and other extensive budget cuts (Witz and Brassil, 2020). It is clear that the COVID-19 pandemic has had an immense impact on college sports and university standings, but it cannot yet be predicted how extensive this impact will be for the upcoming seasons.

It's essential for college athletes to realize that it's not impossible to stay physically connected as a team and prepared for future competitions during the COVID-19 pandemic. Creating meaningful relationships with teammates and leaning on strong leaders when necessary are potential ways for teams to work on their team culture while being physically apart. Mental and physical health are also two notions college athletes should constantly strive to improve, with mental health being notably important during periods of isolation during the pandemic. Using virtual platforms to maintain connections and improving sleep habits could both boost mental health and impact later athletic performance for college athletes. Athletes should also take advantage of online platforms for video feedback and develop visualization techniques to assess physical performance, especially with numerous gyms and other facilities being closed during the pandemic. At the end of the day, the most important things for college athletes at the moment are to stay healthy in every way they can and to look forward to the resumption of competition this spring.

## Author Contributions

AR conceptualized the paper and wrote the first edition of the manuscript. AR and AH contributed to the manuscript with their expertise, read, edited, and approved the submitted version. All authors contributed to the article and approved the submitted version.

## Conflict of Interest

The authors declare that the research was conducted in the absence of any commercial or financial relationships that could be construed as a potential conflict of interest.
